# Associations of ethnicity, skin tone, and genome-wide sequencing with bone mineral density in adolescents

**DOI:** 10.1038/s41390-024-03588-4

**Published:** 2024-10-17

**Authors:** Catherine M. Gordon, Abby F. Fleisch, Marie-France Hivert, Lisa B. Rokoff, Sheryl L. Rifas-Shiman, Jean L. Raphael, Emily Oken

**Affiliations:** 1 Division of Intramural Research, Eunice Kennedy Shriver National Institute of Child Health and Human Development, Bethesda, MD USA; 2 Center for Interdisciplinary Population and Health Research, MaineHealth Institute for Research, Portland, ME USA; 3 Pediatric Endocrinology and Diabetes, Maine Medical Center, Portland, ME USA; 4 Diabetes Unit, Massachusetts General Hospital, Boston, MA USA; 5 Division of Chronic Disease Research Across the Lifecourse, Department of Population Medicine, Harvard Medical School and Harvard Pilgrim Health Care Institute, Boston, MA USA; 6 Department of Pediatrics, Texas Children’s Hospital and Baylor College of Medicine, Houston, TX USA

## Abstract

**Background:**

Dual-energy x-ray absorptiometry reference data designate Black and non-Black categories, as higher BMD has been documented among Black youth. We examined associations of race, skin tone, and genetic factors with bone mineral density (BMD).

**Methods:**

557 adolescents were followed longitudinally. Exposures included race, skin tone, and principal components (PC) from genome-wide arrays. Total body BMD Z-score (BMD-Z) was the primary outcome using linear regression.

**Results:**

359 adolescents identified as non-Hispanic White (NHW) and 75, non-Hispanic Black (NHB). BMD-Z was higher in NHB vs. NHW (β: 0.92 units, 95% CI: 0.64, 1.19) or those with darker skin (0.79, 95% CI: 0.49, 1.08 for brown vs. medium). The first genetic PC (PC1) correlated with identification as NHB. PC1 was associated with higher BMD-Z (0.09, 95% CI: 0.06, 0.12), even after including race (0.07, 95% CI: 0.00, 0.14) or skin tone (0.10, 95% CI: 0.05, 0.15); both race (0.26, 95% CI: −0.49, 1.01 for NHB vs. NHW) and skin tone (−0.08, 95% CI: −0.59, 0.44 for brown vs. medium) no longer predicted BMD-Z after adjustment for PC1.

**Conclusion:**

Genetic similarity was robustly associated with BMD, prompting a reevaluation of adolescent BMD reference data to exclude the consideration of race.

**Impact:**

- Current bone density reference databases include a binary assignment of patients into “Black” and “non-Black” categories, as a higher BMD has been documented among those identifying as Black compared with individuals of other racial and ethnic backgrounds.
- This study found genetic similarity to be more strongly associated with bone density by dual-energy x-ray absorptiometry than race or skin tone.
- These data emphasize a need to reevaluate how bone density measurements are interpreted, including exploring reference data that exclude the consideration of race.

## Introduction

The use of race and ethnicity in clinical decision-making has come under appropriate scrutiny. Although race is a strong predictor of many health outcomes, it is a social construct that has no biological basis.^1,2^ Racial inequities reflect differential experiences and economic, political and social conditions, including racism.^3^ The many concerns raised with using race as an input into clinical algorithms include the fact that there is greater genetic diversity within compared with across racial categories; that it is unclear and inconsistent who determines a patient’s race or ethnicity for clinical care; and that validly assigning individuals to a single racial category is essentially impossible given the diverse demography of many populations.^2^ Perhaps the most significant problem with using race clinically, however, is that it provides fertile ground for racial bias and systemic racism in medical care.^4^ Acknowledging these concerns, expert bodies have recently reevaluated the inclusion of or removed race as a consideration for clinical tools. Recent examples include the calculation of estimated glomerular filtration rate to evaluate kidney function,^5^ algorithms for determining risk for pediatric urinary tract infections,^6^ and reference data for pulmonary function testing.^7^ These race-agnostic approaches have not been found to be inferior to the prior, race-adjusted tools.^6,8^

Bone density references for both children and adults continue to include a binary assignment of patients into the categories of “Black” and “non-Black”.^9–11^ As recently as August 2022, experts published reference ranges for bone mineral content and areal bone mineral density (BMD) assessed by dual x-ray absorptiometry (DXA) in 1- to 5-year-old children, including separate ranges for Black and non-Black children.^12^ These separate reference ranges were derived because higher BMD has been consistently documented among those identifying as Black compared with those of other racial and ethnic backgrounds. Black individuals also have lower rates of osteoporosis and fractures.^13–16^ The association of darker skin tone (or pigmentation) with higher BMD has been shown to extend within strata of specific racial or ethnic groups in limited studies among adults.^17,18^

Like skin tone,^19^ BMD is a trait that is highly heritable.^20^ An understanding of the genetics of osteoporosis has advanced significantly with the emergence of genome-wide association studies (GWAS).^20–22^ While BMD clearly has some genetic basis, it is unclear to what extent the association of Black race or darker skin tone with higher BMD is due to genetics (i.e., sub-Saharan African heritage) versus other factors like environmental or behavioral variables.^23^

Few studies to date have examined these three related characteristics – self-identified race and ethnicity, skin tone, and genetic background – in relation to BMD, and published studies are only among adults. In the current study, we hypothesized that self-identified Black race, darker skin tone, and genetic similarity would each be directly correlated with higher BMD. We also hypothesized that darker skin tone would predict higher BMD even within self-identified White and Black adolescents, and that genetic similarity would be a stronger predictor of BMD than race or skin tone.

## Methods

### Participants

For this secondary analysis, we studied participants in Project Viva, a prospective cohort established to examine associations of pre- and postnatal factors with maternal and child health.^24^ Briefly, we recruited women in early pregnancy 1999–2002 at prenatal visits at Atrius Harvard Vanguard Medical Associates, a large, multispecialty group practice in eastern Massachusetts. We enrolled 2128 live singleton infants and attempted to follow them at periodic research visits after delivery, including during infancy, early childhood, mid-childhood, early adolescence, and mid-adolescence. We also administered annual questionnaires, including one in the year following the mid-adolescent visit (the “Year 19 Questionnaire”). We included in the present analysis the 557 children with information on bone mineral density in mid-adolescence and information on race, ethnicity, and skin tone.

### Ethics statement

The Institutional Review Board of Harvard Pilgrim Health Care approved the project. All mothers provided written informed consent at enrollment and follow-up visits, and all children provided verbal assent if under age 18 or written informed consent if they were above age 18 years at outcome assessment.

### Data collection procedures/assessment of exposures

On the Year 19 Questionnaire, teens self-reported their race and ethnicity in response to the following questions: “What is your race? Select all that apply” and “Are you of Hispanic, Latinx, or Spanish Origin?” If they were missing information on this variable, we assigned them to the category that mothers reported for them in early childhood (*N* = 182). Self-reported and mother-reported race were highly concordant. For our primary measure, we combined race and ethnicity to assign individuals to mutually exclusive categories of non-Hispanic White (NHW), non-Hispanic Black (NHB), non-Hispanic Asian (NHA), or Hispanic. We also included an “other race” group because of small numbers identifying in other categories (including more than one race), but we recognize that this is an especially non-homogenous grouping and therefore do not highlight or separately discuss any results within this category.

At the mid-adolescent visit, teens self-reported their skin tone in 6 categories based on the Fitzpatrick skin types, in response to the question “which of these statements best describes your skin?”.^25^ Response options included: (1) extremely fair skin, always burns, never tans; (2) fair skin, always burns, sometimes tans; (3) medium skin, sometimes burns, always tans; (4) olive skin, rarely burns, always tans; (5) brown skin, never burns, always tans; (6) dark brown skin, never burns, always tans.

We extracted DNA from children’s white blood cells and genotyped it using Illumina Infinium Core Exome-24 microarray chips (Illumina, Inc., San Diego, CA). For whole genome imputation, we used the 1000 G Phase 3 (version 5) on the Michigan Imputation Server (v1.0.4).^26^

We employed the GENESIS package in R to perform principal component analysis (PCA) on our genetic data. PCA is a dimensionality reduction technique that transforms the data into orthogonal components (principal components), ordered by the variance they explain. This process involved standardizing the genetic data matrix, calculating the covariance matrix, and performing eigen decomposition to extract and analyze the principal components.

The differentiation of principal components (PCs) is carried out using a mathematical and objective approach. PCs are identified based on their eigenvalues and their capacity to capture variance, rather than by subjective judgment. In our analysis, the first principal component (PC1) captured the most variance and was strongly associated with self-identified Black race, aligning with prior multi-ethnic population whole-genome PCA studies.^26^ The second principal component (PC2) correlated most strongly with Asian race (eFig. 1), and the third principal component (PC3) with Hispanic ethnicity (eFig. 2). Among the 557 participants included in the current analyses, 401 had high quality genotyping (and derived PCs).

### Assessment of outcomes

Trained and certified research assistants measured total body-less-head BMD using DXA (Discovery A - Hologic Inc., Marlborough, MA).^27^ We used the same DXA scanner for all participants and calibrated it daily. We analyzed data using Hologic software (version 12.6). Intra-rater reliability was high (intraclass correlation = 0.99).

### Assessment of covariates

We calculated age at the mid-adolescent visit as the difference between visit date and birth date. Mothers reported their educational attainment at study enrollment and their marital status, annual household income, and age (calculated from visit date and date of birth) at the mid-adolescent visit.

Also at the mid-adolescent visit, the parent or guardian reported adolescent pubertal development using a 5-item written pubertal development scale.^28–30^ To assign a summary pubertal development score to each participant, we calculated the average score across all items.

Trained research assistants measured adolescents’ weight using a calibrated Tanita scale (model TBF-300A; Tanita Corporation of America, Inc., Arlington Heights, IL) and height using a calibrated stadiometer (Shorr Productions, Olney, MD) using standardized protocols, from which we calculated BMI-for-age-and-sex Z-scores (BMI-Z).^31^

### Statistical analysis

To evaluate the contribution of race, we calculated Z-scores (BMD-Z) in two ways. We calculated “race-agnostic” BMD-Z using U.S. age-, sex-, population ancestry, and height-specific reference data for ages 5–20 years by assigning all participants as non-Black.^9^ In addition, we calculated “race-adjusted” BMD-Z using the same reference data but coding individuals as either Black or non-Black in accordance with their self-identified race.

We described participant characteristics overall and according to self-reported race and ethnicity. In our analyses, we used NHW as the reference category because it was the group with the largest sample size in our cohort. We conducted multivariable linear regression analyses to examine the relationships of each exposure with the race-adjusted and race-agnostic BMD-Z scores. We included the three exposures individually (separate models for each exposure) and together in the same models. We also examined associations of skin pigmentation and genetic PCs with BMD-Z within strata of NHB and NHW.

Our primary analyses were unadjusted, as we were interested in prediction but not causal inference. In sensitivity analyses, we adjusted models for maternal age, education, marital status, and annual household income; adolescent pubertal status and BMI-Z; and adolescent height and sex, which are inputs into reference algorithms. We also limited models examining race and ethnicity or skin pigmentation as exposures to the *n* = 401 who also had genetic PC data.

## Results

Among the 557 participants with mid-adolescent BMD measures, mean (SD) age was 17.6 (0.6) years, race-agnostic BMD-Z was −0.53 (1.15) units, and race-adjusted BMD-Z was −0.70 (1.09) units. The majority (64%) identified as NHW, 13% as NHB, 2% as NHA, 11% as Hispanic, and 9% as more than one race, or other race (Table 1). Just over half (54%) of adolescents were female, 72% had mothers who were college graduates, 82% had mothers who were married or cohabiting, and 70% had annual household income above $100,000. Skin tone tracked with but was not perfectly aligned with race (Table 1 and eTable 1). Those who identified as NHB vs. NHW had higher mean (SD) PC1 values [7.80 (1.48) vs. −1.94 (0.25)] and race-agnostic BMD-Z [0.18 (1.15) vs. −0.74 (1.07)], but race-adjusted BMD-Z were more similar [−0.60 (1.11) vs. −0.74 (1.07)]. As expected, within the stratum of NHW, race-agnostic and race-adjusted results were exactly the same.

**Table 1:** Characteristics of 557 Project Viva adolescents with bone mineral density (BMD) results, overall and according to self-reported race and ethnicity.

	Overall	Non-Hispanic White	Non-Hispanic Black	Non-Hispanic Asian	Hispanic	More than one race or other
Adolescent characteristics	*n* = 557	359 (64%)	75 (13%)	13 (2%)	62 (11%)	48 (9%)
	**Mean (SD) or N (%)**					
Skin Tone, %						
Extremely fair	44 (8%)	38 (11%)	2 (3%)	0 (0%)	3 (5%)	1 (2%)
Fair	165 (30%)	151 (42%)	1 (1%)	1 (8%)	9 (15%)	3 (6%)
Medium	182 (33%)	134 (37%)	7 (9%)	7 (54%)	18 (29%)	16 (33%)
Olive	74 (13%)	33 (9%)	6 (8%)	3 (23%)	17 (27%)	15 (31%)
Brown	74 (13%)	3 (1%)	42 (56%)	2 (15%)	14 (23%)	13 (27%)
Dark Brown	18 (3%)	0 (0%)	17 (23%)	0 (0%)	1 (2%)	0 (0%)
Principal component from genetic data*						
PC1	−0.06 (3.56)	−1.94 (0.25)	7.80 (1.48)	0.35 (0.46)	1.79 (3.67)	1.55 (3.21)
PC2	0.06 (3.75)	−0.95 (0.31)	-1.01 (0.59)	19.51 (6.05)	0.86 (1.95)	2.86 (4.75)
PC3	0.20 (0.67)	−0.06 (0.20)	1.47 (0.86)	−0.35 (0.25)	0.57 (0.68)	0.22 (0.63)
Female, %	299 (54%)	199 (55%)	37 (49%)	9 (69%)	30 (48%)	24 (50%)
Age, years	17.6 (0.6)	17.6 (0.6)	17.6 (0.5)	17.3 (0.3)	17.6 (0.6)	17.5 (0.6)
Race-agnostic BMD-Z	−0.53 (1.15)	−0.74 (1.07)	0.18 (1.15)	−0.50 (1.13)	−0.32 (1.26)	−0.34 (1.08)
Race-adjusted BMD-Z	−0.70 (1.09)	−0.74 (1.07)	−0.60 (1.11)	−0.50 (1.13)	−0.55 (1.19)	−0.76 (1.13)
BMD raw, g/cm^2^	0.97 (0.11)	0.96 (0.10)	1.04 (0.11)	0.95 (0.11)	0.97 (0.11)	0.97 (0.10)
BMI-Z	0.42 (1.05)	0.32 (0.97)	0.86 (1.10)	−0.02 (1.22)	0.71 (1.20)	0.20 (1.03)
Height, cm	170.5 (9.3)	170.7 (9.0)	172.1 (9.1)	168.0 (12.3)	168.3 (10.5)	169.4 (8.5)
Puberty score, 0-4 points scale	3.7 (0.4)	3.7 (0.3)	3.6 (0.4)	3.7 (0.4)	3.7 (0.4)	3.7 (0.4)
**Maternal characteristics**						
College graduate, %	402 (72%)	303 (85%)	34 (46%)	13 (100%)	22 (35%)	30 (63%)
Married or cohabiting, %	436 (82%)	303 (89%)	42 (58%)	13 (100%)	44 (72%)	34 (76%)
Annual household income >$100,000, %	361 (70%)	263 (80%)	29 (41%)	9 (75%)	28 (47%)	32 (73%)
Age, years	50.8 (5.1)	52.0 (3.9)	47.8 (6.8)	50.0 (3.2)	48.2 (6.4)	50.0 (5.9)

^*^Using whole genome data for *N* = 401 participants, we identified principal components (PCs) that identified the greatest variance in genetic architecture in our group of participants. The first PC (PC1) identified the most diverse genetic architecture in the population and correlated strongly with self-identified Black race, the second (PC2) with Asian race, and the third (PC3) with Hispanic ethnicity

In unadjusted analyses, compared with those who identified as NHW, race-agnostic BMD-Z was higher for those who identified as NHB (0.92, 95%CI: 0.64, 1.19), Hispanic (0.42 units, 95% confidence interval [CI]: 0.12, 0.71), or as more than one race or other race (0.39, 95%CI: 0.06, 0.73). There were no significant differences in race-adjusted BMD-Z by self-reported race or ethnicity (Table 2). Skin tone was associated with race-agnostic BMD-Z [(e.g., 1.06, 95% CI: 0.53, 1.60) higher for dark brown (vs. medium skin tone)], whereas associations of skin tone with race-adjusted BMD-Z were weaker and confidence intervals included the null (Table 2). Higher PC1 was associated with race-agnostic BMD-Z (0.09 units, 95% CI: 0.06, 0.12 per each 0.1 increment in PC1), but not race-adjusted BMD-Z (0.01, 95% CI: −0.02, 0.04). PC1 was also associated with higher BMD-Z: 0.07, 95% CI: 0.00, 0.15, after including race, skin tone, and PC1 in the same model (*p* = 0.05). Although PC3 was also associated with greater race-agnostic BMD-Z, in models mutually adjusting for both, only PC1 was independently associated (Table 2). PC2 was not associated with either BMD outcome (Table 2).

**Table 2:** Associations of race and ethnicity, skin tone, and genetic principal components (PCs) with bone mineral density (BMD) Z-scores.

Exposure	Race-agnostic BMD Z-score	Race-adjusted BMD Z-score
	β (95% CI)	β (95% CI)
**Race and ethnicity alone**		
Hispanic	**0.42 (0.12, 0.71)**	0.19 (−0.10, 0.49)
Non-Hispanic White	0.0 (ref)	0.0 (ref)
Non-Hispanic Black	**0.92 (0.64, 1.19)**	0.13 (−0.14, 0.41)
Asian	0.24 (−0.37, 0.85)	0.24 (−0.37, 0.85)
>1 race or other	**0.39 (0.06, 0.73)**	−0.02 (−0.36, 0.31)
**Non-Hispanic Black alone (Yes vs. No)**	**0.82 (0.55, 1.09)**	0.11 (−0.16, 0.37)
**Skin tone alone**		
Extremely fair, always burns, never tans	−0.28 (−0.64, 0.09)	−0.29 (−0.65, 0.07)
Fair, always burns, sometimes tans	0.11 (−0.12, 0.34)	0.16 (−0.07, 0.39)
Medium, sometimes burns, always tans	0.0 (ref)	0.0 (ref)
Olive, rarely burns, always tans	**0.37 (0.07, 0.67)**	0.22 (−0.08, 0.51)
Brown, never burns, always tans	**0.79 (0.49, 1.08)**	0.21 (−0.08, 0.51)
Dark brown, never burns, always tans	**1.06 (0.53, 1.60)**	0.35 (−0.18, 0.88)
Genetic Principal Components (PCs)*		
PC1	**0.09 (0.06, 0.12)**	0.01 (−0.02, 0.04)
PC2	0.00 (−0.04, 0.03)	0.00 (−0.03, 0.03)
PC3	**0.36 (0.19, 0.52)**	0.03 (−0.13, 0.20)
**PC1, PC2, PC3 in same model**		
PC1	**0.11 (0.06, 0.16)**	0.02 (−0.03, 0.07)
PC2	−0.01 (−0.04, 0.02)	0.00 (−0.03, 0.03)
PC3	−0.12 (−0.41, 0.17)	−0.05 (−0.34, 0.23)
**Race and ethnicity and PC1 in same model**		
Hispanic	0.14 (−0.29, 0.57)	0.21 (−0.22, 0.64)
Non-Hispanic White	0.0 (ref)	0.0 (ref)
Non-Hispanic Black	0.26 (−0.49, 1.01)	0.19 (−0.56, 0.93)
Asian	0.12 (−0.61, 0.84)	0.28 (−0.44, 1.00)
>1 race or other	0.23 (−0.24, 0.70)	0.05 (−0.42, 0.52)
PC1	0.07 (0.00, 0.14)	0.00 (−0.07, 0.06)
**Skin tone and PC1 in same model**		
Extremely fair, always burns, never tans	−0.09 (−0.53, 0.34)	−0.11 (−0.54, 0.33)
Fair, always burns, sometimes tans	0.07 (−0.20, 0.35)	0.06 (−0.21, 0.33)
Medium, sometimes burns, always tans	0.0 (ref)	0.0 (ref)
Olive, rarely burns, always tans	0.07 (−0.28, 0.43)	0.02 (−0.33, 0.38)
Brown, never burns, always tans	−0.08 (−0.59, 0.44)	−0.17 (−0.68, 0.34)
Dark brown, never burns, always tans	−0.03 (−0.83, 0.78)	−0.08 (−0.88, 0.73)
PC1	**0.10 (0.05, 0.15)**	0.02 (−0.02, 0.07)

^*^PCs modeled as continuous, per each 0.1 increment in PC.

Bolding indicates where the confidence intervals exclude the null.

Results were essentially identical when we additionally adjusted models for maternal age, education, marital status, and household income (eTables 2 and 3). When we adjusted for adolescent pubertal status and BMI-Z, there was slight attenuation (eTables 2 and 3), but estimates generally had the same direction and significance, except that race-agnostic BMD-Z was no longer significantly different for Hispanic vs. White adolescents. Results were also similar when we restricted analyses for race and ethnicity to the *n* = 401 with genetic information (data not shown).

Lastly, since DXA derived BMD measures are predicted by height, regression models were constructed adjusting for height and sex, and results were not notably different from those shown in Table 2 (data not shown).

In models limited to those who identified as NHB, darker self-reported skin pigmentation remained associated with higher race-agnostic BMD-Z (0.83 units, 95% CI: 0.05, 1.72 for brown and 0.76 units, 95% CI: −0.21, 1.73 for dark brown, each compared with medium skin color). In models limited to those who identified as NHW, lighter self-reported skin tone  was associated with lower race-agnostic BMD-Z (0.29 units, 95% CI: 0.05, 0.54 for fair and 0.43 units, 95% CI: 0.03, 0.83 for olive, each compared with medium skin color). However, PC1 was not associated with BMD-Z within either subset (Table 3).

**Table 3:** Associations of skin tone categories and principal component 1 (PC1) from genome-wide analysis with race-adjusted bone mineral density (BMD) Z-scores at the Mid-Adolescent Visit among those identifying as non-Hispanic Black or non-Hispanic White.

	Medium, sometimes burns, always tan as reference category		Brown, never burns, always tans as reference category for NHB and Fair, always burns, sometimes tans as reference category for NHW	
Exposure	β (95% CI)		β (95% CI)	
	Non-Hispanic Black only	Non-Hispanic White only	Non-Hispanic Black only	Non-Hispanic White only
**Skin tone alone**				
Extremely fair, always burns, never tans	0.08 (−1.66, 1.82)	−0.10 (−0.48, 0.28)	−0.75 (-2.32, 0.82)	**−0.39 (−0.77,−0.02)**
Fair, always burns, sometimes tans	−1.48 (−3.79, 0.84)	**0.29 (0.05, 0.54)**	**-2.31 (-4.50,−0.11)**	0.0 (ref)
Medium, sometimes burns, always tans	0.0 (ref)	0.0 (ref)	−0.83 (-1.72, 0.05)	**−0.29 (−0.54,−0.05)**
Olive, rarely burns, always tans	0.31 (−0.90, 1.52)	**0.43 (0.03, 0.83)**	−0.52 (-1.47, 0.43)	0.14 (−0.26, 0.53)
Brown, never burns, always tans	0.83 (−0.05, 1.72)	1.18 (−0.03, 2.39)	0.0 (ref)	0.88 (−0.33, 2.09)
Dark brown, never burns, always tans	0.76 (−0.21, 1.73)	–	−0.07 (−0.69, 0.55)	–
PC1 alone*	−0.08 (−0.32, 0.16)	−0.16 (−0.66, 0.34)	−0.08 (−0.32, 0.16)	−0.16 (−0.66, 0.34)
Skin tone and PC1* in same model				
Extremely fair, always burns, never tans	0.71 (-1.90, 3.31)	0.10 (−0.36, 0.55)	−0.06 (−2.50, 2.38)	−0.11 (−0.55, 0.34)
Fair, always burns, sometimes tans	−1.57 (-4.18, 1.03)	0.20 (−0.09, 0.49)	−2.34 (−4.78, 0.10)	
Medium, sometimes burns, always tans	0.0 (ref)	0.0 (ref)	−0.77 (−1.95, 0.41)	−0.20 (−0.49, 0.09)
Olive, rarely burns, always tans	0.26 (−1.26, 1.79)	0.27 (−0.19, 0.73)	−0.50 (-1.68, 0.68)	0.07 (−0.39, 0.53)
Brown, never burns, always tans	0.77 (−0.41, 1.95)	−0.05 (−2.16, 2.07)	0.0 (ref)	−0.25 (-2.37, 1.87)
Dark brown, never burns, always tans	0.51 (−0.78, 1.79)	–	−0.26 (-1.16, 0.64)	–
PC1	−0.05 (−0.30, 0.20)	−0.13 (−0.65, 0.39)	−0.05 (−0.30, 0.20)	−0.13 (−0.65, 0.39)

*PCs modeled as continuous, per each 0.1 increment in PC.

Bolding indicates where the confidence intervals exclude the null.

## Discussion

Our findings contribute to continued active discussions about the use of race in understanding bone density results. The empiric use of race in clinical decision making has come under question, but race as a binary variable continues to be used in the prediction and interpretation of BMD for children and adolescents,^12,13^ as well as adults.^32,33^ In this study, we confirmed prior findings that using a race-agnostic reference yields significantly different BMD Z-score results in Hispanic, NHB, and other races vs. NHW adolescents, whereas there were no significant differences when using the race-adjusted reference. We observed that lighter skin tone was associated with lower BMD-Z even in stratified analyses within both NHB and NHW youth. Thus, some population variation in expected BMD remains even after accounting for a binary “Black” and “non-Black” categorization. Lastly, using principal components from genome-wide analysis, we derived the first axis of genetic similarity that captures the most diverse genetic architecture (PC1) and showed that individuals identifying as NHB had a higher PC1 value. A higher PC1 predicted BMD-Z even after inclusion of either race or skin tone, whereas neither race nor skin tone predicted BMD-Z after adjustment for PC1, suggesting that genetic similarity is a more robust predictor of BMD-Z than race or skin tone.

There are numerous reasons why incorporating race, especially a “Black” vs. “non-Black” binary, in bone density reference data is problematic. First, as we demonstrated not all of the difference in the expected BMD is captured by these two categories. Also, genetic diversity is greater within people of African descent compared with across differing racial groups.^34–36^ Given the importance of genetics in explaining variance in BMD (i.e., genetic differences typically explain 70-80% of peak bone mass),^37^ a simple binary division would not be able to capture this variation.

Moreover, the current race-adjusted BMD Z-score suggests that an individual who self-identifies as Black should have a different BMD threshold for clinical intervention than an individual who self-identifies as non-Black. However, there are little to no data on whether the association between BMD and fracture risk differs by race, skin tone, or genetic similarity, independent of other risk factors. In other words, if any given absolute BMD value is equally associated with fracture risk regardless of an individual’s race (or skin pigmentation or genetics), then there is no clinical utility in adjusting BMD reference data for race. Even if race provides additional information in determining the relationship between BMD and fracture risk, it is likely that race itself may not account for this difference but is merely a marker for other, unmeasured risk factors that predispose to fracture, such as environmental exposures, physical activity, or diet.^37^

Further considerations include that use of race-adjusted references puts someone – the patient, DXA technologist, clinician, or maybe an administrator at a hospital or health plan – into the uncomfortable position of assigning an individual (e.g., with diverse racial background) to a category that may not be appropriate or a “fit.” This empiric determination is also likely to be highly inaccurate,^38,39^ especially for those with darker skin. Also, directly cueing clinical staff and providers to consider race enforces the idea of “superior” and “inferior” groups.^40^ “Socially assigned” race has been shown to be a stronger predictor of health-care related discrimination than self-identified race.^41^

Our findings build on previous reports emphasizing the importance of genetics in explaining the variance in BMD.^37,42,43^ The GWAS approach has helped to delineate the underpinnings of bone mass.^44^ For example, the CPED1-WNT16-FAM3C locus plays a signature role in BMD trajectory,^22^ and a recent report identified 65 candidate genes linked to bone development.^45^ However, more research is needed to build on the suggestion that genetic variants that predict either low or higher bone density are more common within certain populations.^46^ Indeed, genetic variants predicting lower BMD have been found in greater frequency in individuals of European vs. African descent.^46^ For example, SNP rs2536195-A (within RMBS3) has been identified in 60% of individuals of European descent, but only 14% of those of African descent.^47^ Similarly, genetic variants predicting higher BMD have been found in greater frequency in those with African vs. European backgrounds.^47^

Even beyond racial categories, skin tone has implications for health that primarily reflect lived experience rather than any biological difference. Black Americans with dark vs. lighter skin have substantially worse outcomes for a range of health metrics, including mortality.^48^ It is likely that nuanced social experiences of Blacks with different observed skin tone markedly change the experience of racial inequality.

One apparent solution would be to account for genetic similarity or regional ancestry in the BMD Z-score rather than race. Our results showing PC1 to be a robust predictor of BMD than racial category or skin tone also support this potential approach. However, genetic ancestry information is not clinically available and, moreover, is inexplicably connected with skin tone and with characteristics of appearance that track with various racial groups. Thus, even though genetic variants are biological determinants, genetic similarity (as captured in PC1) ultimately also reflects the same differences in experience and economic, political, and social conditions as skin tone or race.^3^

Our study has several limitations. The sample size was relatively small, resulting in wide confidence intervals for some comparisons and prohibiting stratified analyses within some groups. Participants all had prenatal care and insurance at enrollment, and all were from the same geographic area, limiting generalizability. Nevertheless, we enrolled participants from a range of racial identities that reflected the diversity of the source population.^24^ We were not able with the current design to adjust for certain confounding variables such as environmental and lifestyle factors; thus the findings may oversimplify the relationships between studied exposures and BMD. Data on skin tone were obtained by self-report with its inherent limitations, although the Fitzpatrick classification is the most widely accepted method of skin phototyping.^25^ We did not have contemporaneous 25-hydroxyvitamin D measurements. However, since circulating vitamin D levels would be lower with darker skin pigmentation, it does not explain and actually makes more complex the relationship between race or skin tone with BMD. We did not collect information on fractures, which would further the clinical significance of the current findings. We obtained BMD measurements of the whole body, composed of primarily cortical bone. Future studies should also include additional skeletal sites such as the hip and lumbar spine, which would allow for an evaluation of the relationships of race, skin tone, and genetics with both cortical and trabecular-rich sites. Lastly, we acknowledge that any binary categorization of BMD-Z does not fully capture the complexity and diversity within and across racial and ethnic groups. Nonetheless, we attempted to minimize the effect of race by the generation of a “race-agnostic” measure, recognizing that while providing new insights, it could be viewed as another oversimplification of a complex issue.

## Conclusion

In summary, there are demonstrated harms associated with use of race in clinical decision making and uncertain benefits from its inclusion in bone density reference values. Future work is needed to evaluate clinical guidelines and policies to avoid embedded racism. Use of race as a factor within practice guidelines can lead clinicians to miss opportunities to address racism-related causes of a disease such as osteoporosis or make false assumptions about risk factors, which could potentially impact treatment decisions. Our results suggest that while genetics may play a role in BMD, race – a social construct with no biological basis – does not. We hope that the current findings will serve as a stimulus to recognize the problems associated with the use of race and ethnicity when evaluating both fracture and future osteoporosis risk, as well as drawing conclusions about scientific data related to bone health. A binary categorization of this variable is not able to capture the complexity and diversity within and across racial and ethnic groups, and other approaches need to be explored.

## Supplementary information

Supplementary Material

## Data Availability

The datasets generated during and/or analysed during the current study are available from the corresponding author on reasonable request.
