# Supplementary material for: Associations of ethnicity, skin tone, and genome-wide sequencing with bone mineral density in adolescents
Source: Pediatr Res. 2024 Oct 17;97(7):2376–82. doi: 10.1038/s41390-024-03588-4 (PMC12003700; doi:10.1038/s41390-024-03588-4)

**Supplementary Material:**

**eTable 1:** Distributions of participant characteristics according to self-reported skin tone

**eTable 2:** Adjusted associations of race and ethnicity, skin tone, and genetic principal components with **race-agnostic** bone mineral density (BMD) Z-scores

**eTable 3:** Adjusted associations of race and ethnicity, skin tone, and genetic principal components with **race-adjusted** bone mineral density (BMD) Z-scores

**eFigure 1:** The distributions of principal components 1 and 2 (PC1 and PC2) are shown from genome-wide analysis, according to self-reported race and ethnicity.

**eFigure 2:** The distributions of principal components 1 and 3 (PC1 and PC3) are shown from genome-wide analysis, according to self-reported race and ethnicity.

**eTable 1:** Distributions of participant characteristics according to self-reported skin tone

|  |  | **Skin tone** | | | | | |
| --- | --- | --- | --- | --- | --- | --- | --- |
|  | **Overall** | **Extremely fair** | **Fair** | **Medium** | **Olive** | **Brown** | **Dark brown** |
|  | n=557 | 44 (8%) | 165 (30%) | 182 (33%) | 74 (13%) | 74 (13%) | 18 (3%) |
|  | **Mean (SD) or N (%)** | | | | | | |
| Race and ethnicity, % |  |  |  |  |  |  |  |
| . Hispanic | 62 (11%) | 3 (7%) | 9 (5%) | 18 (10%) | 17 (23%) | 14 (19%) | 1 (6%) |
| . Non-Hispanic White | 359 (64%) | 38 (86%) | 151 (92%) | 134 (74%) | 33 (45%) | 3 (4%) | 0 (0%) |
| . Non-Hispanic Black | 75 (13%) | 2 (5%) | 1 (1%) | 7 (4%) | 6 (8%) | 42 (57%) | 17 (94%) |
| . Asian | 13 (2%) | 0 (0%) | 1 (1%) | 7 (4%) | 3 (4%) | 2 (3%) | 0 (0%) |
| . >1 race or other | 48 (9%) | 1 (2%) | 3 (2%) | 16 (9%) | 15 (20%) | 13 (18%) | 0 (0%) |
| *Principal component from GWAS data** |  |  |  |  |  |  |  |
| PC1: Mean (SD) | -0.06 (3.56) | -1.21 (2.67) | -1.74 (1.14) | -1.21 (2.04) | 0.47 (3.48) | 5.95 (3.18) | 8.42 (0.56) |
| PC2: Mean (SD) | 0.06 (3.75) | -0.81 (0.84) | -0.66 (2.21) | 0.60 (5.01) | 1.06 (4.43) | -0.01 (2.71) | -1.02 (0.41) |
| PC3: Mean (SD) | 0.20 (0.67) | 0.09 (0.65) | -0.01 (0.46) | 0.00 (0.34) | 0.24 (0.71) | 1.14 (0.78) | 1.32 (0.78) |
| Race-agnostic BMD Z-score | -0.53 (1.15) | -1.00 (1.03) | -0.62 (1.00) | -0.73 (1.17) | -0.36 (1.21) | 0.06 (1.13) | 0.34 (0.90) |
| Race-adjusted BMD Z-score | -0.70 (1.09) | -1.07 (1.05) | -0.63 (1.01) | -0.79 (1.14) | -0.57 (1.19) | -0.58 (1.08) | -0.44 (0.88) |
| BMD raw (g/cm^3^) | 0.97 (0.11) | 0.92 (0.10) | 0.96 (0.10) | 0.96 (0.10) | 0.98 (0.11) | 1.03 (0.10) | 1.06 (0.12) |

*Using whole genome data for N=401 participants, we identified principal components (PCs) that identified the greatest variance in genetic architecture in our group of participants. The first PC (PC1) identified the most diverse genetic architecture in the population and correlated strongly with self-identified Black race, the second (PC2) with Asian race, and the third (PC3) with Hispanic ethnicity.

**eTable 2:** Adjusted associations of race and ethnicity, skin tone, and genetic principal components with **race-agnostic** bone mineral density (BMD) Z-scores

|  | **Unadjusted** | **Adjusted for maternal age, education, household income, marital status** | **Adjusted for teen mid-adolescent pubertal status and BMI z-score** |
| --- | --- | --- | --- |
| **Exposure** | **β (95% CI)** | | |
| **Race and ethnicity alone** |  |  |  |
| . Hispanic | **0.42 (0.12, 0.71)** | **0.42 (0.09, 0.75)** | 0.27 (-0.02, 0.55) |
| . Non-Hispanic White | 0.0 (ref) | 0.0 (ref) | 0.0 (ref) |
| . Non-Hispanic Black | **0.92 (0.64, 1.19)** | **0.95 (0.63, 1.26)** | **0.73 (0.47, 1.00)** |
| . Asian | 0.24 (-0.37, 0.85) | 0.38 (-0.26, 1.02) | 0.38 (-0.19, 0.96) |
| . >1 race or other | **0.39 (0.06, 0.73)** | **0.44 (0.08, 0.79)** | **0.46 (0.14, 0.78)** |
| **Non-Hispanic Black alone (Yes vs. No)** | **0.82 (0.55, 1.09)** | **0.78 (0.48, 1.08)** | **0.64 (0.38, 0.90)** |
| **Skin tone alone** |  |  |  |
| . Extremely fair, always burns, never tans | -0.28 (-0.64, 0.09) | -0.27 (-0.66, 0.12) | -0.23 (-0.58, 0.11) |
| . Fair, always burns, sometimes tans | 0.11 (-0.12, 0.34) | 0.07 (-0.18, 0.31) | 0.11 (-0.12, 0.33) |
| . Medium, sometimes burns, always tans | 0.0 (ref) | 0.0 (ref) | 0.0 (ref) |
| . Olive, rarely burns, always tans | **0.37 (0.07, 0.67)** | 0.29 (-0.02, 0.61) | 0.23 (-0.06, 0.51) |
| . Brown, never burns, always tans | **0.79 (0.49, 1.08)** | **0.74 (0.41, 1.06)** | **0.66 (0.37, 0.94)** |
| . Dark brown, never burns, always tans | **1.06 (0.53, 1.60)** | **0.90 (0.33, 1.47)** | **0.91 (0.39, 1.42)** |
| **Genetic Principal Components (PCs) alone*** |  |  |  |
| PC1 | **0.09 (0.06, 0.12)** | **0.09 (0.06, 0.13)** | **0.08 (0.05, 0.11)** |
| PC2 | 0.00 (-0.04, 0.03) | 0.00 (-0.03, 0.04) | 0.00 (-0.03, 0.03) |
| PC3 | **0.36 (0.19, 0.52)** | **0.30 (0.11, 0.50)** | **0.30 (0.14, 0.46)** |
| **PC1, PC2, PC3 in same model** |  |  |  |
| PC1 | **0.11 (0.06, 0.16)** | **0.11 (0.06, 0.17)** | **0.09 (0.04, 0.14)** |
| PC2 | -0.01 (-0.04, 0.02) | 0.00 (-0.04, 0.03) | 0.00 (-0.03, 0.03) |
| PC3 | -0.12 (-0.41, 0.17) | -0.15 (-0.45, 0.15) | -0.09 (-0.36, 0.18) |
| **Race and ethnicity and PC1 in same model** |  |  |  |
| . Hispanic | 0.14 (-0.29, 0.57) | 0.17 (-0.28, 0.63) | 0.09 (-0.32, 0.49) |
| . Non-Hispanic White | 0.0 (ref) | 0.0 (ref) | 0.0 (ref) |
| . Non-Hispanic Black | 0.26 (-0.49, 1.01) | 0.19 (-0.59, 0.97) | 0.12 (-0.57, 0.82) |
| . Asian | 0.12 (-0.61, 0.84) | 0.28 (-0.49, 1.05) | 0.31 (-0.36, 0.99) |
| . >1 race or other | 0.23 (-0.24, 0.70) | 0.24 (-0.24, 0.73) | 0.30 (-0.15, 0.74) |
| PC1 | 0.07 (0.00, 0.14) | **0.07 (0.00, 0.15)** | **0.06 (0.00, 0.13)** |
| **Skin tone and PC1 in same model** |  |  |  |
| . Extremely fair, always burns, never tans | -0.09 (-0.53, 0.34) | -0.05 (-0.52, 0.41) | -0.18 (-0.59, 0.24) |
| . Fair, always burns, sometimes tans | 0.07 (-0.20, 0.35) | 0.06 (-0.23, 0.35) | 0.04 (-0.22, 0.30) |
| . Medium, sometimes burns, always tans | 0.0 (ref) | 0.0 (ref) | 0.0 (ref) |
| . Olive, rarely burns, always tans | 0.07 (-0.28, 0.43) | 0.03 (-0.34, 0.40) | -0.01 (-0.34, 0.33) |
| . Brown, never burns, always tans | -0.08 (-0.59, 0.44) | -0.09 (-0.63, 0.45) | 0.03 (-0.46, 0.51) |
| . Dark brown, never burns, always tans | -0.03 (-0.83, 0.78) | -0.23 (-1.09, 0.63) | -0.03 (-0.81, 0.74) |
| PC1 | **0.10 (0.05, 0.15)** | **0.10 (0.05, 0.16)** | **0.08 (0.03, 0.12)** |

*PCs modeled as continuous, per each 0.1 increment in PC

**eTable 3:** Adjusted associations of race and ethnicity, skin tone, and genetic principal components with **race-adjusted** bone mineral density (BMD) Z-scores

|  | **Unadjusted** | **Adjusted for maternal age, education, household income, marital status** | **Adjusted for teen mid-adolescent pubertal status and BMI z-score** |
| --- | --- | --- | --- |
| **Exposure** | **β (95% CI)** | | |
| **Race and ethnicity alone** |  |  |  |
| . Hispanic | 0.19 (-0.10, 0.49) | 0.27 (-0.06, 0.59) | 0.06 (-0.22, 0.34) |
| . Non-Hispanic White | 0.0 (ref) | 0.0 (ref) | 0.0 (ref) |
| . Non-Hispanic Black | 0.13 (-0.14, 0.41) | 0.21 (-0.10, 0.52) | -0.04 (-0.31, 0.22) |
| . Asian | 0.24 (-0.37, 0.85) | 0.37 (-0.27, 1.00) | 0.38 (-0.19, 0.95) |
| . >1 race or other | -0.02 (-0.36, 0.31) | 0.06 (-0.29, 0.41) | 0.04 (-0.28, 0.36) |
| **Non-Hispanic Black alone (Yes vs. No)** | 0.11 (-0.16, 0.37) | 0.13 (-0.17, 0.42) | -0.07 (-0.32, 0.19) |
| **Skin tone alone** |  |  |  |
| . Extremely fair, always burns, never tans | -0.29 (-0.65, 0.07) | -0.27 (-0.66, 0.11) | -0.23 (-0.57, 0.12) |
| . Fair, always burns, sometimes tans | 0.16 (-0.07, 0.39) | 0.11 (-0.14, 0.35) | 0.16 (-0.06, 0.38) |
| . Medium, sometimes burns, always tans | 0.0 (ref) | 0.0 (ref) | 0.0 (ref) |
| . Olive, rarely burns, always tans | 0.22 (-0.08, 0.51) | 0.15 (-0.16, 0.46) | 0.08 (-0.20, 0.37) |
| . Brown, never burns, always tans | 0.21 (-0.08, 0.51) | 0.22 (-0.10, 0.54) | 0.09 (-0.20, 0.37) |
| . Dark brown, never burns, always tans | 0.35 (-0.18, 0.88) | 0.25 (-0.31, 0.81) | 0.21 (-0.30, 0.72) |
| **Genetic Principal Components (PCs) alone*** |  |  |  |
| PC1 | 0.01 (-0.02, 0.04) | 0.02 (-0.02, 0.05) | 0.00 (-0.03, 0.03) |
| PC2 | 0.00 (-0.03, 0.03) | 0.01 (-0.02, 0.04) | 0.01 (-0.02, 0.04) |
| PC3 | 0.03 (-0.13, 0.20) | 0.03 (-0.16, 0.21) | -0.02 (-0.18, 0.14) |
| **PC1, PC2, PC3 in same model** |  |  |  |
| PC1 | 0.02 (-0.03, 0.07) | 0.03 (-0.03, 0.08) | 0.00 (-0.05, 0.05) |
| PC2 | 0.00 (-0.03, 0.03) | 0.01 (-0.02, 0.04) | 0.01 (-0.02, 0.04) |
| PC3 | -0.05 (-0.34, 0.23) | -0.08 (-0.37, 0.22) | -0.03 (-0.30, 0.25) |
| **Race and ethnicity and PC1 in same model** |  |  |  |
| . Hispanic | 0.21 (-0.22, 0.64) | 0.26 (-0.20, 0.71) | 0.16 (-0.25, 0.56) |
| . Non-Hispanic White | 0.0 (ref) | 0.0 (ref) | 0.0 (ref) |
| . Non-Hispanic Black | 0.19 (-0.56, 0.93) | 0.11 (-0.67, 0.89) | 0.05 (-0.65, 0.75) |
| . Asian | 0.28 (-0.44, 1.00) | 0.43 (-0.34, 1.19) | 0.47 (-0.20, 1.15) |
| . >1 race or other | 0.05 (-0.42, 0.52) | 0.07 (-0.41, 0.55) | 0.12 (-0.33, 0.56) |
| PC1 | 0.00 (-0.07, 0.06) | 0.00 (-0.07, 0.08) | -0.01 (-0.07, 0.06) |
| **Skin tone and PC1 in same model** |  |  |  |
| . Extremely fair, always burns, never tans | -0.11 (-0.54, 0.33) | -0.07 (-0.53, 0.40) | -0.19 (-0.60, 0.22) |
| . Fair, always burns, sometimes tans | 0.06 (-0.21, 0.33) | 0.05 (-0.24, 0.33) | 0.03 (-0.23, 0.29) |
| . Medium, sometimes burns, always tans | 0.0 (ref) | 0.0 (ref) | 0.0 (ref) |
| . Olive, rarely burns, always tans | 0.02 (-0.33, 0.38) | -0.03 (-0.40, 0.35) | -0.06 (-0.39, 0.28) |
| . Brown, never burns, always tans | -0.17 (-0.68, 0.34) | -0.17 (-0.71, 0.36) | -0.07 (-0.56, 0.41) |
| . Dark brown, never burns, always tans | -0.08 (-0.88, 0.73) | -0.29 (-1.15, 0.56) | -0.08 (-0.86, 0.70) |
| PC1 | 0.02 (-0.02, 0.07) | 0.03 (-0.02, 0.09) | 0.01 (-0.04, 0.05) |

*PCs modeled as continuous, per each 0.1 increment in PC

**eFigure 1:** Distributions of principal component 1 (PC1) and PC2 from genome-wide analysis.


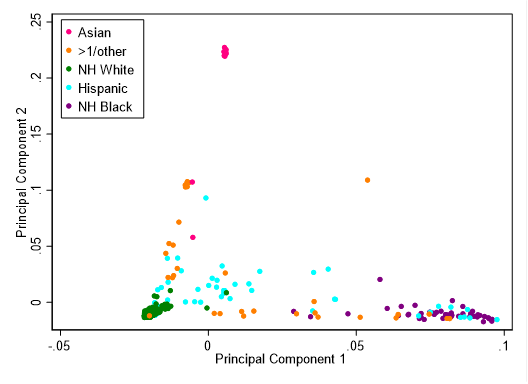


**eFigure 2:** Distributions of principal component 1 (PC1) and PC3 from genome-wide analysis.


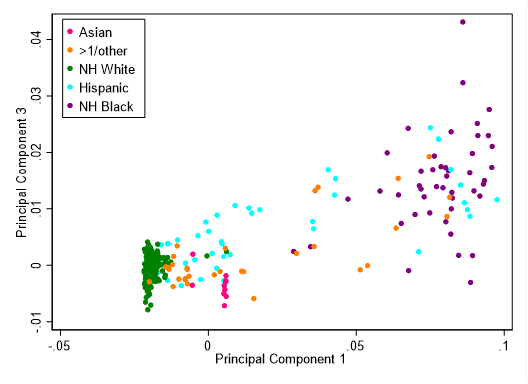

Supplement: Supplementary file 1 — Supplementary Material [file 41390_2024_3588_MOESM1_ESM.docx]
